# Coronavirus in Colombia: Stigma and quarantine

**DOI:** 10.7189/jogh.10.020372

**Published:** 2020-12

**Authors:** Ana M Trejos-Herrera, Stefano Vinaccia, Marly J Bahamón

**Affiliations:** 1 Psychology Department, Universidad del Norte, Barranquilla, Colombia; 2 Psychology Department, Research Group I-flor. Universidad del Sinú, Montería, Colombia; 3 Psychology Department, Universidad Simón Bolívar, Barranquilla, Colombia

This study describes a brief history of the spread of coronavirus, listing the disease’s main characteristics. The decisions made by the Colombian government are presented, as there have been progressively more cases of infection, death and recovery, in addition to the stigmatisation that the medical personnel who are treating COVID-19 patients have experienced from some people in Colombia. Issues related to intra-family and domestic partner violence occurring in Colombia because of the quarantine processes are also addressed. Early stress studies in general population and medical professionals. The lines of work being implemented by the Colombian Association of Psychology (COLPSIC, for its Spanish acronym) for psychological help towards the affected population are described. Finally, psychological intervention initiatives that are important to implement for mental well-being are discussed.

## CORONAVIRUS

Severe acute respiratory syndrome 2 (SARS-CoV-2), coronavirus, originated in bats and was transmitted to humans through unknown intermediary animals in Wuhan, Hubei Province, China in December 2019 [1]. The disease is transmitted through the process of inhalation or by coming into contact with infected droplets, and the incubation period ranges from 2 to 14 days. Many people are asymptomatic. The fatality rate is estimated to be 2-3% [2]. Symptoms usually include fever, cough, and sore throat, shortness of breath, fatigue and general discomfort, among others. The effects of the disease are mild in most people. In some (generally the elderly and those with pre-existing conditions), it can progress to pneumonia, acute respiratory distress syndrome and multiple organ dysfunction [3]. According to Report by the World Health Organization on coronavirus COVID-19, 24 092 709 cases of this virus and 824 194 deaths have been confirmed. This situation exposes a serious global problem that has had profound repercussions on the health of human beings, because in addition to all the medical implications it entails, the measures taken to prevent possible outbreaks that would exceed the health system’s capacity to respond result in drastic changes to everyone’s lifestyle. Preventive isolation, as well as the constant threat to the survival of the human race, leads people to experience extreme emotional states that can seriously affect their mental and emotional health.

## COLOMBIA

The COVID-19 pandemic was first reported in Colombia on 6 March 6 2020, with the first confirmed case in the country being a 19-year-old woman. On 20 March, a complete quarantine for 19 days was declared in the country to prevent the spread of the virus. On 21 March, the first death was confirmed, and on 6 April, the quarantine was extended up to April 27. Subsequently there were two extensions of the quarantine until 31 August. On August 26, Colombia had 592 128 positive cases and 17 889 deaths.

In this regard, it is necessary to consider the multiple consequences of this situation, especially when the rates of poverty and employment and quality of life among Colombians are not the most favourable for shutting down the country’s economy. According to the Colombian National Administrative Department of Statistics (DANE, for its Spanish acronym) in 2018, 19.6% of the country’s population lived in a situation of multidimensional poverty [4].

## STIGMATISING INCIDENTS

Stigma is a complex process that results from the interaction of stereotypes, prejudice and discrimination. When applied to health conditions, stigma can contribute to a lack of recovery and resources, as well as self-devaluation. People with stigmatised health problems may feel too embarrassed to seek treatment and others may lead them to having fewer opportunities [5].

In Colombia, during the pandemic, the stigma has been transferred to health personnel, which has translated into behaviours such as general refusal to use public transportation services, hostility towards neighbours who refuse to share any type of space and annoyance with customers and employees for entering grocery stores, among other disobedient behaviours, which have even escalated to violent acts, such as punching and threatening anyone who wears a uniform disguising themselves as a member of the health care sector. These events led Colombian President Ivan Duque to publicly suggest the possibility of fining those who participate in this type of behaviour. On 31 March, the Colombian Paediatric Society issued a formal complaint against these acts involving discrimination, rejection and eviction from their homes.

## QUARANTINE

Mandatory quarantine or isolation is the separation and restriction of movement of persons who may have been exposed to a contagious disease to determine if they become ill, thus reducing the risk of infecting others [6].

Modern quarantine strategies have been imposed around the world in an attempt to reduce the spread of COVID-19 in different countries, including short- and medium-term closures, voluntary curfews for households, restricting group gatherings, cancelling planned social and public events, closing public transportation systems and other travel restrictions [7].

Various research studies [8-10] have shown that one of the most important protective factors for mental health is social support, a paradoxical issue while in isolation, because isolation has specifically been studied as a risk factor, particularly for conditions linked to depression and suicide [11]. Thus, the psychological effects of quarantine include emotional disturbance, stress-induced depression, bad moods, irritability, insomnia, stress and posttraumatic stress symptoms [12].

The possible effects on the mental and emotional health of Colombians in relation to the COVID-19 health emergency are still uncertain. However, incidents of domestic violence, gender violence, depression, suicide, acute stress and many other issues are expected to increase. Some research has reported a tendency to experience symptoms of posttraumatic stress, as well as an exacerbation of depression and anxiety [13-15]. Their partners are reported to have killed 110 women between March and July.

## DISTRESS STUDIES

A study conducted by Profamilia [16] of 3549 people in 10 cities in Colombia with high and low coronavirus circulation showed that 75% of those surveyed had had some mental health problem related to the mandatory preventive isolation decreed by the Covid-19 pandemic. “Of this 75% who claim to have had some mental illness during quarantine, 54% say they felt nervous, 53% tired, 46% impatient and 3% felt anger or rage about the isolation, with young people between 18 and 29 years most affected by the situation.

**Figure:**
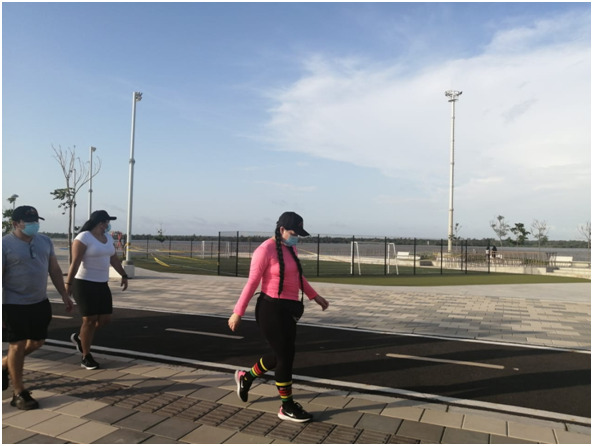
Photo: Barranquilla citizens walking and exercising in quarantine at the Tourist Boardwalk front Rio Magdalena, Barranquilla, Colombia (from the authors collection, used with permission).

On the other hand, Monterrosa [17] in an exploratory research with a sample of 531 medical doctors working on Colombian territory. 215 (40%) male doctors and 316 (59.6%) female doctors. They studied the levels of distress. In the responses obtained, half of the general practitioners reported extreme tiredness or exhaustion and 40% reported inability to sleep, headaches and headaches, strong temptations to not get up in the morning or gastrointestinal discomfort. Twenty percent said they had shortness of breath or a choking feeling, decreased appetite, nervous muscle tremors, painful sensations in different parts of the body, sweating and palpitations.

More recently in Colombia, a study was developed to estimate the high risk of suicide during COVID-19 confinement in the Colombian population700 adults between 18 and 76 years of age (M = 37.1, SD = 12.7; 68.0% women) completed an online questionnaire. The results showed that 7.6% of the participants reported a high risk of suicide. The high risk of suicide was associated with a high perception of COVID-19 related stress, risk of depressive episode and insomnia. The study further suggested that 1 in 13 Colombians in a non-probability sample reported a high risk of suicide during COVID-19 [18].

Finally, a study was developed to evaluate the prevalence and variables related to the perceived stress associated with the COVID-19 pandemic in a sample of Colombian adults through a cross-sectional survey designed online. Adults answered a version of the Perceived Stress Scale (PSS-10) modified for COVID-19 (PSS-10-C), with Cronbach alpha equal to 0.86. In total, 406 individuals aged between 19 and 88 years (M = 43.9; SD = 12.4) agreed to participate in the survey: 61.8% were females, 90.6% had a university degree, 44.1% were health professionals, and 45.7% considered public health policies for preventing the spread of the disease inconsistent with scientific recommendations. PSS-10-C scores ranged from 0 to 36 (M = 16.5; SD = 7.3); 58 individuals (14.3%) scored for high perceived stress (cut-off point = 25). The inconsistency between policies and scientific evidence was significantly related to high perception of stress associated with COVID-19 (odds ratio (OR) = 2.36; 95%CI = 1.32-4.20), after adjusting for gender. We concluded that the study group presented the prevalence of perceived stress associated with COVID-19 at high levels, arising from the inconsistent strategies developed by health authorities in view of scientific recommendations [19].

## COLOMBIAN ASSOCIATION OF PSYCHOLOGY

The Colombian College of Psychology (COLPSIC, for its Spanish acronym) came up with different initiatives to manage the psychological crisis associated with COVID-19. The first includes supporting the AMIGA telephone line in handling crises, the second provides emotional support for health personnel, the third develops information programmes for the general community, and the fourth advises the media to ensure that psychological information in the area is based on evidence.

## DISCUSSION

In less than five months, a large part of the world’s population, particularly the Colombian population, has stepped away from its normal rhythm of life into unprecedented, prolonged periods of confinement and social distancing because of the COVID-19 pandemic. Moving forward, it is important to assume that anxiety, despair and stress will increase in a significant portion of the Colombian population when this pandemic ends. In this regard, the phenomenon of red rags that during the quarantine put on the facades of Colombian families to report that they are hungry describes very well the relationship between pandemic and poverty [20]. It will be necessary to develop resilience programmes to implement safeguards for the mental well-being of Colombians. Considering the current conditions and the inability to meet face-to-face, interventions mediated by technology constitute a useful alternative that has proven to be effective in addressing mental health problems [21].

Finally, as suggested by Horesh and Brown [22], clinical intervention programmes should be developed to address the following key areas related to COVID-19: a) diagnosis, b) prevention, c) public communication and dissemination, d) working with medical personnel and incorporating non-mental health services and e) researching specific trauma due to COVID-19.

## References

[1] Singhal T A review of coronavirus disease-2019 (COVID-19). Indian J Pediatr. 2020;87:281-6. 10.1007/s12098-020-03263-6 32166607 PMC7090728

[2] Li L Chen M Critical patients with coronavirus disease 2019. Risk factors and outcome nomogram. J Infect. 2020;80:e37-8. 10.1016/j.jinf.2020.03.025 32272120 PMC7194672

[3] Huang X Wei F Hu L Wen L Chen K Epidemiology and clinical characteristics of COVID-19. Arch Iran Med. 2020;23:268-71. 10.34172/aim.2020.09 32271601

[4] DANE. Pobreza monetaria y multidimensional en Colombia, 2018. Available: https://www.dane.gov.co/index.php/estadisticas-por-tema/pobreza-y-condiciones-de-vida/pobreza-y-desigualdad/pobreza-monetaria-y-multidimensional-en-colombia-2018. Accessed: 11 July 2020.

[5] Corrigan PW Nieweglowski K How does familiarity impact the stigma of mental illness? Clin Psychol Rev. 2019;70:40-50. 10.1016/j.cpr.2019.02.001 30908990

[6] Centers for Disease Control and Prevention. Quarantine and isolation. 2019. Available: https://www.cdc.gov/quarantine/index.html. Accessed: 11 July 2020.

[7] Usher K Bhullar N Jackson D Life in the pandemic: Social isolation and mental health. J Clin Nurs. 2020;29:2756-7. 10.1111/jocn.15290 32250493

[8] Azpiazu L Esnaola I Sarasa M Capacidad predictiva del apoyo social en la inteligencia emocional de adolescentes. Eur J Educ Psychol. 2015;8:23-9. 10.1016/j.ejeps.2015.10.003

[9] Tahmasbipour N Taheri A. A survey on the relation between social support and mental health in students Shahid Rajaee University. Proc Soc Beh Sci. 2012;47:5-9. 10.1016/j.sbspro.2012.06.603

[10] Trejos-HerrerA A Bahamón M Vélez J Alarcón-Vásquez Y Validity and reliability of the Multidimensional Scale of Perceived Social Support in Colombia. Psych Interv. 2018;27:56-63. 10.5093/pi2018a1

[11] Bahamón J Alarcón-Vásquez Y Trejos A Vinaccia S Cabezas A Sepúlveda-Aravena J Efectos del programa CIPRES sobre el riesgo suicida en adolescentes. Rev Psicopatol Psicol Clin. 2019;24:83-91. 10.5944/rppc.23667

[12] Brooks SK Webster RK Smith LE Woodland L Wessely S Greenberg N The psychological impact of quarantine and how to reduce it: Rapid review of the evidence. Lancet. 2020;395:912-20. 10.1016/S0140-6736(20)30460-8 32112714 PMC7158942

[13] Garfin DR Thompson RR Holman EA Acute stress and subsequent health outcomes: A systematic review. J Psychosom Res. 2018;112:107-13. 10.1016/j.jpsychores.2018.05.017 30097129

[14] Lima CKT Medeiros P Silva I Oliveira J Steves J De Souza R Rolim M The emotional impact of coronavirus 2019-nCoV. Psychiatry Res. 2020;28.7. 10.1016/j.psychres.2020.112915 32199182 PMC7195292

[15] Duan L Zhu G Psychological interventions for people affected by the COVID-19 epidemic. Lancet Psychiatry. 2020;7:300-2. 10.1016/S2215-0366(20)30073-0 32085840 PMC7128328

[16] Profamilia. Personal communication. 2020.

[17] Monterrosa APersonal communication. 2020.

[18] Caballero-Domínguez CC Jimenez MP Campo-Arias A Suicide risk during the lockdown due to coronavirus disease (COVID-19) in Colombia. Death Stud. 2020;1-6. Online ahead of print. 10.1080/07481187.2020.1784312 32589519

[19] Pedrozo JC Pedrozo MJ Campo-Arias A Estrés percibido relacionado con la epidemia de COVID-19 en Colombia: una encuesta en línea. Cad Saude Publica. 2020;36:e00090520. 32490918 10.1590/0102-311x00090520

[20] López W, Velandia A. Pandemia y aislamiento en tiempos de desigualdad: las banderas rojas de la cuarentena. Bulletin SIP. 2020;33-36.

[21] Horesh D Brown AD Traumatic stress in the age of COVID-19: A call to close critical gaps and adapt to new realities. Psychol Trauma. 2020;12:331-5. 10.1037/tra0000592 32271070

[22] Vázquez FL Torres Á Blanco V Otero P Hermida E Intervenciones psicológicas administradas por teléfono para la depresión: Una revisión sistemática y meta-análisisTelephone-administered. Rev Ibero Psicol y Sal. 2015;6:39-52. 10.1016/S2171-2069(15)70005-0

